# Human Oncogenic Viruses: Characteristics and Prevention Strategies—Lessons Learned from Human Papillomaviruses

**DOI:** 10.3390/v16030416

**Published:** 2024-03-08

**Authors:** Luisa Galati, Maria Vincenza Chiantore, Mariarosaria Marinaro, Paola Di Bonito

**Affiliations:** 1 Department of Experimental Oncology, IEO, European Institute of Oncology IRCCS, 20139 Milan, Italy; luisa.galati@ieo.it; 2 Department of Infectious Diseases, Viral Hepatitis and Oncovirus and Retrovirus Diseases (EVOR) Unit, Istituto Superiore di Sanità, Viale Regina Elena 299, 00161 Rome, Italy; mariavincenza.chiantore@iss.it; 3 Department of Infectious Diseases, Microorganisms and Host Response: Research and Technological Innovation (MICROS) Unit, Istituto Superiore di Sanità, Viale Regina Elena 299, 00161 Rome, Italy; mariarosaria.marinaro@iss.it

**Keywords:** hepatitis B virus (HBV), hepatitis C virus (HCV), human papillomavirus (HPV), Epstein-Barr virus (EBV; HHV-4), Kaposi sarcoma-associated herpesvirus (KSHV; HHV-8), human immunodeficiency virus-1 (HIV), human T-cell lymphotropic virus type 1 (HTLV-1), oncoviruses

## Abstract

Approximately 12% of human cancers worldwide are associated with infectious agents, which are classified by the International Agency for Research on Cancer (IARC) as Group 1 within the agents that are carcinogenic to humans. Most of these agents are viruses. Group 1 oncogenic viruses include hepatitis C virus, hepatitis B virus (HBV), human T-cell lymphotropic virus type 1, Epstein-Barr virus, Kaposi sarcoma-associated herpesvirus, human immunodeficiency virus-1 and high-risk human papillomaviruses (HPVs). In addition, some human polyomaviruses are suspected of inducing cancer prevalently in hosts with impaired immune responses. Merkel cell polyomavirus has been associated with Merkel cell carcinoma and included by the IARC in Group 2A (i.e., probably carcinogenic to humans). Linking viruses to human cancers has allowed for the development of diagnostic, prophylactic and therapeutic measures. Vaccination significantly reduced tumours induced by two oncogenic viruses as follows: HBV and HPV. Herein, we focus on mucosal alpha HPVs, which are responsible for the highest number of cancer cases due to tumour viruses and against which effective prevention strategies have been developed to reduce the global burden of HPV-related cancers.

## 1. Introduction

Over the past few decades, there have been global efforts to develop preventive and diagnostic tools as well as therapeutic strategies to control infections associated with some human cancers. This review focuses on the general characteristics of human oncogenic viruses, classified by the International Agency for Research on Cancer (IARC) monograph as Group 1, with a particular emphasis on mucosal alpha HPV genotypes. The current knowledge of HPV biology, the natural history and epidemiology of HPV infections, the screening programmes and the available vaccines will be summarised.

## 2. Infectious Agents and Cancer

According to the IARC, approximately 12% (2,300,000 new cases) of global cancers in 2020 were attributable to infectious agents including bacteria, viruses and parasites, with a different geographical distribution between low- and high-income countries [1,2,3]. Following epidemiological and biological findings, the bacterium *Helicobacter pylori*; the hepatitis B virus (HBV); hepatitis C virus (HCV); twelve mucosal high-risk human papillomaviruses (known as HR HPVs); Epstein-Barr virus (EBV), which is also known as human herpesvirus 4 (HHV4); Kaposi sarcoma-associated herpesvirus (KSHV), which is also known as human herpesvirus type 8 (HHV-8); human T-cell lymphotropic virus type 1 (HTLV-1); and the parasites *Opisthorchis viverrini*, *Clonorchis sinensis* and *Schistosoma haematobium* were evaluated and classified by the IARC monograph programme as Group 1 biological agents (i.e., carcinogenic to humans) [4]. Despite its indirect carcinogenic role, HIV-1 is classified as Group 1 due to the increased risk of cancer as a consequence of viral-induced immune suppression in people living with HIV (PLWHIV) [5,6,7,8]. Indeed, PLWHIV are at risk of coinfections with oncogenic viruses, with the consequence being that related cancers frequently arise in PLWHIV such as those linked to HPV (e.g., cervical and anal cancers), EBV (Hodgkin and non-Hodgkin lymphomas) and HHV-8 (Kaposi sarcoma) infections [9,10,11]. In the last update, the bacterium *H. pylori* (*n* = 850,000, 36.3%) and viruses such as the HR HPV genotypes (*n* = 730,000, 31.1%), HBV (*n* = 380,000, 16.4%) and HCV (*n* = 170,000, 7.4%) were reported as the most prevalent pathogens responsible for infection-related cancers [2]. Of note, approximately 64% of cancers attributable to infections were related to viruses in 2018, namely HPV, HBV, HCV and EBV [2]. Therefore, several public health measures have been globally implemented to prevent and control infection-related cancers. The evidence that some cancers are linked to specific viral infections has led to the development of preventive, diagnostic tools and also therapeutic measures in some clinical settings. These measures have all contributed to reduce the incidence of virus-related cancers in some regions of the world. For instance, HPV-related cervical cancers have been reduced through vaccination, screening programmes and the treatment of HPV-related premalignant cervical lesions [3,12,13,14]. In addition, a reduction of hepatocellular carcinoma (HCC) cases linked to hepatitis virus infection has been observed following vaccination against HBV and through the use of antiviral drugs against HCV [15].

## 3. Oncogenic Viruses

Oncogenic viruses are classified into six different viral families, namely *Retroviridae*, *Papillomaviridae*, *Polyomaviridae*, *Flaviviridae*, *Hepadnaviridae* and *Herpesviridae* (Table 1). These viruses are specific to humans and have either a DNA or RNA genome. Viruses with a DNA genome include EBV, KSHV, HPV and HBV, while those with a RNA genome include HCV, HIV and HTLV-1. Some oncogenic viruses are associated with a single tumour type, while others are associated with multiple types of cancer, which may arise at different anatomical sites (Table 1). For instance, HPV-related cancers can develop in different areas of the body, as indicated in Table 1. The outcome of a viral infection depends on the viral type and host factors, which involve complex interactions between the virus and the host [16].

The RNA tumour viruses induce cellular transformation and immortalization through different mechanisms [17]. As is the case for animal retroviruses, they usually carry oncogenes of cellular origin (e.g., *Src* in the avian Rous sarcoma virus, RSV) [18] or interfere with cellular oncogenes (e.g., pro-virus integration proximal to a cellular oncogene or a tumour suppressor) [17,19]. Alternatively, retroviruses can promote cellular transformation by expressing accessory proteins able to activate cellular genes that promote proliferation or prevent apoptosis (e.g., the Tax protein from HTLV). Among retroviruses, HTLV-1 is the only virus oncogenic to humans. The well-known oncogenic RNA viruses belong to the *Retroviridae* family, although exceptions exist since HCV belongs to the *Flaviviridae* family. HCV indirectly exerts its oncogenic effect, mainly causing inflammation, fibrosis and cirrhosis with a significantly increased risk of developing HCC. In addition, the expression of viral proteins that have oncogenic potential has been reported and may also increase the risk of developing HCC [20].

The DNA tumour viruses belonging to Group 1 include heterogeneous viruses, as summarised in Table 1. These viruses are conventionally classified by their genome size in (i) small (e.g., HPV) and (ii) large DNA tumour viruses (e.g., EBV). In these viruses, the activity of some viral proteins, which are usually necessary for viral replication, can interfere with and subvert crucial cellular pathways, which may lead to cancer development. Specifically, viral proteins with transforming activity (oncoproteins) can impact cellular proteins such as p53 and pRB family members, whose tumour suppressor activity is essential for preventing cellular transformation.

As reported above for HCV, HBV, which is instead a DNA tumour virus, mainly acts through an indirect carcinogenic mechanism by inducing chronic inflammation and liver damage. However, HBV also expresses proteins with transforming activity, such as HBx, which contribute to its persistence and may further increase the risk of developing HCC [17,20].

Cellular tropism, genome organization and the associated neoplasia of both human DNA and RNA oncogenic viruses included in Group 1 are summarised in Table 1.

**Table 1:** Group 1 cancer-related viruses are listed and divided by virus family, virus name, genome type and size, cellular tropism, associated neoplasia and cancer cases. The percentage of cancer cases is based on 2,300,000 worldwide oncogenic infections with Group 1 pathogens (updated 2020), available from GLOBOCAN, the global cancer observatory and reference [2].

Virus Family	Virus	Year of Discovery	Genome kb	Tropism	Associated Neoplasia	Cases *n* (%)
*Papillomaviridae*	HPV16, 18, 16, 18, 31, 33, 35, 39, 45, 51, 52, 56, 58, 59	1983 [21]	dsDNA 8.0 kb	Keratinocytes	Cervical, anal, vulva, vagina, penis and head and neck cancers	730,000 ^1^ (31.1%)
*Hepadnaviridae*	HBV	1965 [22]	Partial dsDNA 3.3 kb	Hepatocytes	Hepatocellular carcinoma	380,000 ^1^ (16.4%)
*Flaviviridae*	HCV	1989 [23]	Positive-sense RNA strand 9.6 kb	Hepatocytes	Hepatocellular carcinoma and other non-Hodgkin lymphomas	170,000 ^1^ (7.4%)
*Herpesviridae*	EBV (HHV-4)	1964 [24]	dsDNA 172 kb	Epithelial cells and B cells	Hodgkin lymphoma, Burkitt lymphoma and nasopharyngeal carcinoma	156,600 ^2^ (6.8%)
*Herpesviridae*	KSHV (HHV-8)	1994 [25]	dsDNA 140 kb	Epithelial cells and B cells	Kaposi sarcoma	42,000 ^2^ (1.8%)
*Retroviridae*	HTLV-1	1980 [26]	Positive-sense RNA strand 9.0 kb	T and B cells	Adult T-cell leukaemia and lymphoma	3600 ^2^ (0.16%)

^1^ Number of attributable cases derived from GLOBOCAN 2020 (https://gco.iarc.fr, accessed on 15 November 2023). ^2^ Number of attributable cases derived from [2].

Although animal polyomaviruses (PyVs) can cause tumours in their natural hosts or in animal models, the role of HPyVs in human cancer is still under investigation since cancer mainly arises in immunocompromised hosts [27,28]. The Merkel cell polyomavirus (MCPyV) is a naked double-stranded DNA virus with a small genome (5.4 kb), which replicates in epidermal keratinocytes and dermal fibroblasts. MCPyV is abundantly detected in healthy-looking skin. The virus is ubiquitous and results in asymptomatic infections in healthy subjects, and its exposure can occur early in life [29,30]. The virus is present in the healthy skin of various body sites, and it is clonally integrated in about 80% of Merkel cell carcinomas (MCCs), where viral small and large T antigens (sT and LT) are persistently expressed [31,32,33]. The MCC is an aggressive neuroendocrine tumour originating from Merkel cells, which are part of the tactile-end organs in the skin. Cancer mainly arises in skin exposed to UV and in older adults [32]. The IARC classifies the MCPyV as group 2A (probably carcinogenic to humans), whereas the the BK polyomavirus (BKPyV) and the John Cunningham polyomavirus (JCPyV) are classified as group 2B (possibly carcinogenic to humans) [32,34].

Group 1 human oncogenic viruses, despite their biological and structural differences, share common traits. Firstly, they are a necessary but not the only cause leading to cancer development in the infected host. Secondly, they establish persistent infections and have evolved strategies to evade host immune responses, which are crucial for clearing viral infections. The outcome of the infection depends on the host immune system. Indeed, some viral-related cancers arise prevalently in the context of host immunosuppression, chronic inflammation or host genetic background [7,17,35,36]. Cancers usually arise in persistently infected hosts even decades after the acute primary infection. Some viral infections (i.e., EBV, HPV) can be acquired during a person’s lifetime and can be frequent in the general population. Fortunately, the incidence of their related tumours is low.

Viral oncogenesis is a “rare” and accidental event occurring during a long-term infection that does not promote viral replication or viral fitness but often results in a “dead-end” for the infecting virus [35,37]. For instance, in HPV-related cervical cancers, the viral genome is integrated into the host genome, thus leading to a non-productive infection since part of the viral genome is lost during integration, thereby preventing the virus to complete its life cycle.

**Figure 1:**
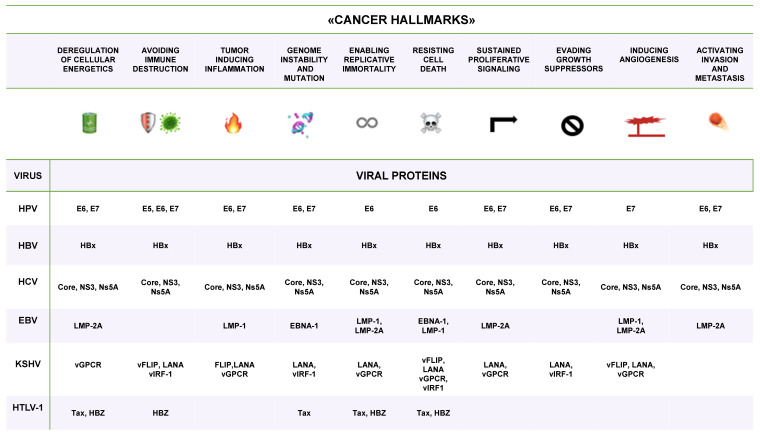
“Cancer hallmarks” according to Hanahan and Weinberg. The oncoproteins of each virus are listed in the column of the corresponding cancer hallmarks. The information is summarised from [40,41,42,43].

Oncogenic viruses can induce cancer by reprogramming cellular pathways through the activity of viral oncoproteins [38]. These proteins impair essential cell pathways by targeting key host proteins that control various cellular functions. Table 2 shows the main proteins targeted by the viral oncoproteins. Different viruses target the same proteins (Table 2) [39]. A transformed cell acquires functional abilities and sustains cellular growth, evasion of cell death, cellular immortalization and angiogenesis, it and loses capabilities involved in DNA repair and cell cycle regulation, thereby favouring the accumulation of DNA damage, cellular transformation and neoplastic progression. Functional alterations of transformed cells are known as the “hallmarks of cancer” described by Weinberg and Hanahan [40,41]. These hallmarks [40,41] are described in the context of the transformation induced by oncogenic viruses (Figure 1) through their proteins [42]. Different viruses can target the same proteins and cellular pathways [39,40,41,42,43].

Cancer is a multi-factorial disease in which the role of host factors (e.g., immune status) [43,44] or exposures to other carcinogens may increase the risk of cellular transformation [17,35,37,45,46].

Host immunity plays a role in determining whether cancer develops after infection with an oncogenic virus, as demonstrated by the increased incidence of virus-induced cancers in immunocompromised individuals. For example, Kaposi sarcoma (KS) was a rare tumour before the HIV/AIDS pandemic, which contributed to increase the KS rate a thousand-fold [47].

Viruses induce a robust immune response, thereby activating receptors of innate immunity that stimulate the production of interferons (IFNs) as first-line responses and later activating adaptive immunity. Innate immune responses are triggered through different pathogen recognition receptors (PRRs) including Toll-like receptors (TLRs), several cytoplasmic sensors for viral DNA (e.g., cGAS) and RNA (e.g., RIG-I and MDA-5). These receptors act through convergent signalling cascades, culminating in the activation of transcription factors that induce the expression of pro-inflammatory cytokines (e.g., IL-1a, IL-1b, IL-18, IL-6 and TNFa) and type I and type III IFNs, as well as IRF3 and IRF7 mainly by interferon response factor (IRF) activation [48]. Viruses also have evolved strategies to escape both innate and adaptive immune host responses, a mechanism known as immune evasion.

Host genetic factors can also influence the clearance and outcome of infections. A genome-wide association study investigated the role of some host genetic variants (e.g., HLA region) in genetic susceptibility to some viral-induced cancers such as HPV-driven cervical cancers [49,50,51]. Establishing a viral aetiology for a tumour is a difficult task that requires extensive studies. Molecular mechanisms of oncogenicity, in vitro and in vivo, and large-scale epidemiological studies are necessary to obtain statistically reliable data to establish the cause-and-effect principle.

Studies on oncogenic viruses have highlighted key pathways and mechanisms conserved in non-viral tumours, which have contributed to a better understanding of human cancer.

## 4. Human Papillomaviruses

The association between certain HPV genotypes and human cancers was discovered about 40 years ago by Harald zur Hausen, who was awarded the Nobel Prize in 2008 for medicine [4,5]. Human papillomaviruses are small (52–55 nm) DNA tumour viruses belonging to the *Papillomaviridae* family. So far, more than 200 HPV genotypes have been phylogenetically classified according to the nucleotide sequence relatedness of the encoded major capsid protein L1 [52,53] into alpha, beta, gamma, mu and nu genera, and they have been numbered in different species (www.hpvcenter.se, accessed on 12 December 2023). Up to now, the genus alpha includes 65 HPV genotypes, while the beta and gamma genera include 54 and 102 types, respectively. The mu genus groups three HPV genotypes, while only one is included in the nu genus (www.hpvcenter.se, accessed on 12 December 2023) [54]. HPVs are heterogenous viruses displaying distinct tropism for mucosal (i.e., alpha HPV genotypes) and cutaneous squamous epithelia (i.e., beta, gamma, mu, nu and some alpha HPV genotypes).

**Figure 2:**
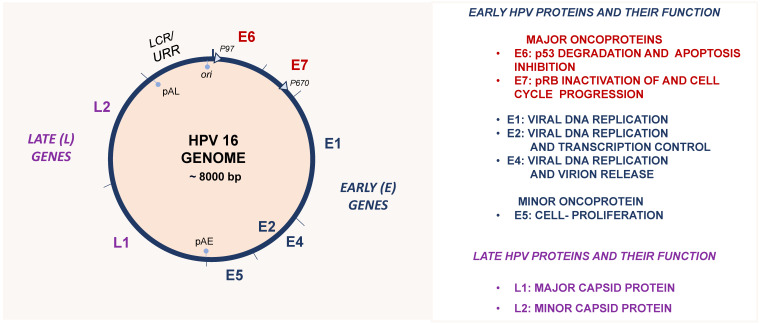
Genome organization of the prototype HPV16 and the main functions of HPV’s early (E) and late (L) encoded proteins. The circular dsDNA genome is indicated by a blue circle in which the six early (E1 up to E7) and the two late (L1 and L2) ORFs are separated by bars. The long control region (LCR), which is also called the URR, contains the origin of replication (ori) and transcription including transcription enhancer sequences. The early (p97) and late (p670) promoters are indicated by light-blue arrows. The early (pAE) and late (pAL) polyadenylation signals are also indicated.

HPVs are non-enveloped viruses with an icosahedral capsid enclosing a double-stranded DNA (dsDNA) genome. The prototype HPV16 genome, which is about 8000 bp in size, consists of (i) an early (E) region that encodes regulatory proteins; is transcribed in the order of E6, E7, E1, E2, E4 and E5; and is involved in viral oncogenesis, transcription, replication and virion release. It also consists of (ii) a late (L) region that encodes the structural major (L1) and minor (L2) viral capsid proteins and (iii) a long control region (LCR), which is also called the upstream regulatory region (URR), as well as a non-coding region containing all the regulatory elements for transcription and the origin (ori) of viral replication (Figure 2) [52].

Despite the well-conserved general structure of their viral genome, HPV genotypes belonging to different genera show different features in terms of genome length and ORFs. For instance, beta and gamma HPV genotypes lack the E5 ORF, while some gamma genotypes (i.e., HPV101, 103 and 108) lack the ORF E6 [55,56,57].

The HR HPV early proteins E5, E6 and E7 have been recognised as the minor (E5) and the major viral oncoproteins (E6 and E7) since they play a key role in promoting carcinogenesis by targeting and inactivating essential cellular proteins [58].

E5 of the prototype HPV16 is a small protein composed of 83 amino acids. It is an integral membrane protein that can self-polymerize in vitro and in vivo to form oligomers. E5 shares the characteristics of a group of viral membrane proteins called viroporins, which are involved in channel activity and the permeabilization of the cell membranes to ions and small molecules [59]. E5 is found in the nuclear and cellular membranes (in the endoplasmic reticulum (ER) and the Golgi apparatus); therefore, it has a central role in altering lipid and protein trafficking inside the cell [60,61]. E5 directly activates the signalling pathways of the epidermal growth factor (EGF)/receptor (EGFR) and upregulates the G protein-coupled endothelin receptor (ETA)/ET1 as well as COX-2 expression, thereby affecting the pathways involved in cell proliferation, angiogenesis, anti-apoptosis and energy metabolism. In the ER, E5 interferes with MHC (Major Histocompatibility Complex) class I and MHC class II protein trafficking, thus contributing to immune evasion [61]. HPV16 oncogenes have been studied in several transgenic mouse models [62]. E5 increases the burden and severity of an E6- and E7-induced tumour, and when expressed alone in the basal layer of the stratified squamous epithelia, it induces hyperplasia, aberrant differentiation and spontaneous skin tumours in transgenic mice. In addition, in the transgenic mouse’s cervical epithelium, E5 induces cancer only after long-term oestrogen treatment [62]. In cervical squamous cell carcinoma (SCC) samples, the levels of E5 transcripts are variable since the E5 gene can be lost during viral integration into the host genome. In cervical intraepithelial neoplasia (CIN) lesions, E5 transcripts are low and are undetectable in OPCs [63,64].

HPV16 E6 and E7 are small (158 and 98 amino acids, respectively) non-enzymatic proteins that interact with various cellular proteins, including tumour suppressor factors p53 and pRB, thereby mediating their inactivation [57,65,66]. The HR HPV early proteins E6 and E7 can impact the cell cycle, DNA repair, apoptosis and differentiation processes, thus favouring the accumulation of chromosomal abnormalities and leading the infected cell towards malignant transformation [66,67,68]. The expression of HPV16 E6 and E7 efficiently immortalize human keratinocytes in vitro [68]. P53 is a transcription factor playing a pivotal role in preventing carcinogenesis by the activation of DNA damage responses (DDRs), cell cycle arrest and apoptosis [69,70,71,72]. The viral protein E6 binds p53 through the cellular ubiquitin E3 ligase protein E6AP (E6-associated protein). Once the E6/E6AP and p53 complex is assembled, the latter is rapidly ubiquitinated and consequently degraded through the proteasome pathway. This determines a loss of the p53 tumour suppressor activity with the consequent alteration of cellular pathways and a progression towards a malignant phenotype of the infected cells [68]. Interestingly, the E6 protein of HR HPVs induces p53 degradation, while the E6 from low-risk (LR) types does not [73].

So far, different host proteins targeted by E6 and involved in cellular proliferation, senescence, apoptosis, immune response and differentiation processes have been identified. E6 is also involved in the activation of the human ribonucleoprotein telomerase reverse transcriptase (hTERT), which is responsible for telomere repeat sequence elongation [74,75,76]. hTERT is usually overexpressed in cancer cells, while its activity is maintained at low levels in normal cells, thereby determining a telomere shortening following each cellular division [76]. In HR HPV-infected cells, hTERT activation can favour telomere length maintenance with each cellular division and promote indefinite cellular proliferation [77,78]. HPV16 E6 promotes the activation of hTERT transcription through the degradation of NFX1-91, which is a repressor of the hTERT promoter [79]. Alternatively, in HR HPV-infected cells, E6 mediates hTERT transcription activation through the recruitment of Myc to the hTERT promoter, thus driving the expression of c-Myc-responsive genes [77]. Conversely, the E6 protein from LR types does not activate hTERT [80].

In addition, the HR HPV E6 early proteins target other host proteins, such as the cellular PDZ proteins, which are named accordingly for their PSD-90/Dlg/ZO-1 (PDZ) homology domain [81,82,83]. The PDZ proteins are important cytoplasmic adapter proteins that participate in the regulation of signalling, trafficking and the function of G protein-coupled receptors. E6 is able to interact with a high number of PDZ proteins, altering important regulators of signal transduction, cell–cell contact and epithelial cell polarity, which might contribute to disease progression [83,84].

The major HPV oncoprotein E7 is an acidic phosphoprotein that binds through the N-terminus motif Leu-X-Cys-X-Glu (LXCXE), the tumour suppressor retinoblastoma (pRb1) and the related members of the pRB family, the pocket proteins p107 and p130 [85]. The pRb protein has a central role in cell cycle control through the negative regulation of the G1 to S transition during cell cycle division. In HR HPV-infected cells, the interaction between the E7 and pRb protein leads to its degradation through the ubiquitin–proteasome pathway. This subsequently activates E2F transcription factors (E2F1–3) with the progression into the S phase of the cell cycle, deregulation of cellular proliferation and expression of pro-proliferative factors. Moreover, the E7 protein is able to bind many other cellular proteins, thereby determining a loss of cell cycle regulation and leading to structural chromosome instability, such as abnormal centrosome numbers [44,86,87].

Overall, the main characteristic of HPV-driven cancers is that of the continuous expression of E6 and E7, which is required for the establishment and maintenance of the malignant phenotype [88]. To carry this out, a persistent HPV infection is essential to progress towards malignancy, and both E6 and E7 are able to overcome the inhibitory effects of IFNs by targeting interferon regulatory factors (IRFs) to evade the host immune response. More specifically, among the IRF family members inactivated by HPV16, IRF-3 activity is impaired by E6, while IRF-1 and IRF-9 are inhibited by E7, as reviewed in [89]. Moreover, E6 and E7, either alone or in combination, are able to inhibit other important cellular pathways to escape the host immune system (e.g., cGAS-STING) [90,91,92].

## 5. Epidemiology and Natural History of HPV Infection

HPV infection can be asymptomatic, subclinical or produce clinical manifestations ranging from benign warts to precancerous lesions and invasive cancer in the persistently infected host. A role of HPV in human carcinogenesis has been established for the mucosal alpha HR genotypes of HPV16, 18, 31, 33, 35, 39, 45, 51, 52, 56, 58 and 59 in cervical, anal, vulvar, penile, vaginal and a subset of oropharyngeal cancers (OPCs). Additionally, the mucosal alpha HPV6 and HPV11 have been epidemiologically classified as LR types according to their prevalent association with benign lesions such as ano-genital warts, which are the most common clinical manifestations of HPV infection worldwide [89]. HPV6 and 11 are rarely associated with cancer [93] and are classified by the IARC monograph into group 3 since they “are unclassifiable as to carcinogenicity to humans” [4].

Almost all cases of cervical and anal squamous cell carcinomas are attributable to HPVs, while the reported fraction of vulvar and penile cancers related to HPV is lower [2,10,94,95,96,97]. Globally, HR HPVs are responsible for approximately 4.5% of human cancers [1].

The most important HPV-related cancer still remains that of cervical cancer (CC), which is responsible for approximately 80% of worldwide HPV-driven cancers, with the highest incidence rate in low-income countries [2,3,4]. Worldwide differences in CC incidence between high- and low-income countries depend on the coverage rates of vaccination and cervical cytology screening strategies adopted for its control. Nevertheless, an increasing prevalence of anal cancer and OPCs is observed in some developed countries [98,99]. Considering global HPV-related head and neck squamous cell carcinoma (HNSCC), approximately 31% of OPC cases are attributable to HR HPV types, while a lower fraction (about 2%) of cancers arising in other head and neck sites (e.g., oral cavity, larynx) is HPV-related [1], as reviewed in [100].

**Figure 3:**
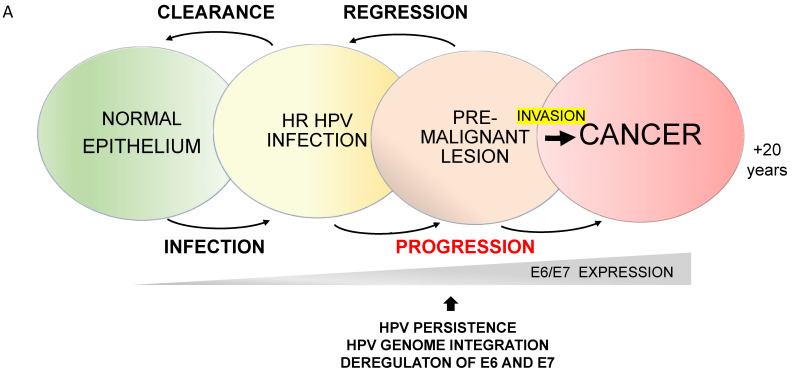

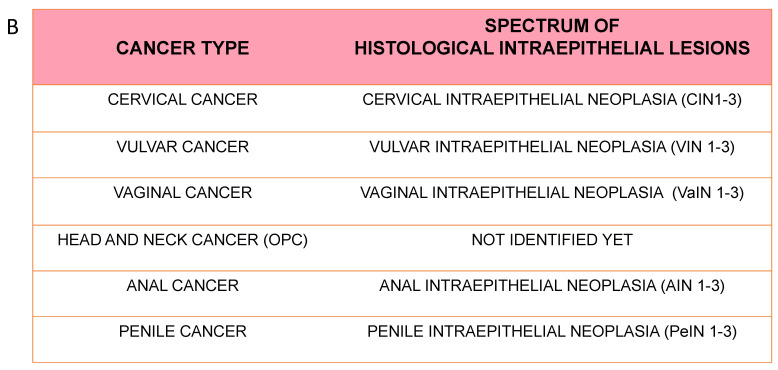
Natural History of HR HPV infection. (**A**) Schematic representation of the progression of HR HPV infection. The host immune system usually clears the infection within a couple of years, and the associated lesion can regress; however, less frequently, the HPV infection can persist and lead to the dysregulation of E6 and E7, which may favour the progression of a premalignant lesion to an invasive cancer. (**B**) Panel reports the spectrum of HPV-related histological premalignant lesions and cancer types occurring in women and men.

Regarding the HPV genotype distribution, HPV16 and HPV18, which are the most oncogenic types, are responsible for more than 70% of CC cases. Indeed, HPV16 is responsible for about 60% of CC cases, while HPV18 contributes to approximately 15% of cases [101]. Notably, HPV16 is also the most frequent genotype detected in other cancers such as anal, penile, vulvar, vaginal and OPCs. HPV infection is among the most prevalent sexually transmitted infections worldwide, with the majority of infections (70% to 90%) cleared by the host immune response within 1 to 2 years. [102]. As already reported, the majority of HR HPV infections are asymptomatic or can result in the development of transient lesions, which are rapidly eliminated by the host immune system. In this condition, the viral life cycle is completed, leading to a productive infection with virion progeny production, while HPV infection is not cleared by the host immune response and may become persistent only in a minority of cases (up to 10%), thereby leading to the development of pre-malignant lesions (Figure 3). These premalignant lesions may then regress or progress towards invasive carcinoma [102,103] (Figure 3). The impairment of host immune responses facilitates HPV persistence and carcinogenesis.

It is important to note that HPV persistency may lead to accidental HPV DNA integration into the host genome [104], thereby determining a failure of the productive viral life cycle and promoting carcinogenesis through the dysregulation of E6 and E7 expression [91]. The integration of HPV DNA can determine the loss of some viral genes (e.g., E1 and E2) [51]. For instance, HPV DNA can be integrated into the host genome with the disruption of the E2 gene, which is a transcriptional repressor of E6 and E7, and this leads to the deregulation of E6 and E7, which in turn promotes carcinogenesis [91]. The loss of the E1 viral gene could give a selective growth advantage and promote clonal expansion [104]. Viral DNA integration along the host genome can occur randomly, at common fragile sites [105] and in transcriptionally active regions [101,106], thereby resulting in the perturbation of cellular homeostasis. In tumour cells, the viral genome can also be detected in an episomal state or coexist in both the episomal and integrated states. The analysis of head and neck cancer samples from the Cancer Genome Atlas programme has also suggested the presence of hybrid viral–human circular episomes [107]. When HPV is only present in an episomal form in cancer, the genome has acquired genetic or epigenetic changes. For instance, methylation of the E2 binding sites in the E6 and E7 promoter determines the deregulation of E6 and E7 [108]. HPV integration rates increase with the severity of the cervical lesions, being higher than 80% in CC and lower in other HPV-related cancers [51,52]. The HPV DNA integration rate is high in some genotypes, and HPV18 has been found to be integrated in 100% of cases [51].

## 6. Prevention Strategies

Vaccination is the primary prevention strategy for HPV-related cervical cancers [109]. Since 2006, HPV vaccines have been licensed and administered through different programmes in several countries to adolescent girls, and they have then been extended to young males. Prophylactic vaccines against some mucosal HPV types consist of the recombinant viral capsid L1 protein, which self-assembles into genome-free virus-like particles (VLPs) when expressed in eukaryotic cells [100]. Immunization with L1-VLP-based vaccines is able to induce serum-neutralizing antibodies, thus preventing viral infection [109]. The prophylactic HPV vaccines licensed worldwide are as follows: (i) a bivalent vaccine (Cervarix^®^, GlaxoSmithKline, Wavre, Belgium) that targets the genotypes HPV16 and HPV18; (ii) a quadrivalent vaccine (Gardasil^®^, Merck & Co, Rahway, NJ, USA) against HPV6, HPV11, HPV16 and HPV18; and (iii) a nonavalent (Gardasil 9^®^, Merck & Co, Rahway, NJ, USA) vaccine that targets nine HPV genotypes, namely HPV6, HPV11, HPV16, HPV18, HPV31, HPV33, HPV45, HPV52 and HPV58. They all resulted in being effective in reducing ano-genital viral infections as well as pre-malignant and malignant lesions [110,111,112,113,114,115].

In order to make vaccines more accessible and affordable in low- and middle-income countries (LMICs), new preventive HPV vaccines have been developed to increase vaccine coverage rates and reduce the costs, thus overcoming barriers and favouring CC elimination [116]. For example, the Cecolin HPV vaccine against HPV16 and HPV18 contains L1-VLPs produced in *Escherichia coli*, and it is licensed in China and a few other countries. The second-generation vaccine Cecolin 9 is an *E. coli*-produced nine-valent HPV L1-VLP vaccine (against genotypes HPV6, HPV11, HPV16, HPV18, HPV31, HPV33, HPV45, HPV52 and HPV58) under clinical trials in China [117]. Another vaccine, the CERVAVAC^®^ HPV vaccine, is a quadrivalent HPV vaccine against HPV 6, HPV11, HPV16 and HPV18, which is developed and manufactured by the Serum Institute of India (SIIPL). It contains L1-VLPs produced in *Hansenula polymorpha* yeast and was approved in India in 2023 [118].

**Figure 4:**
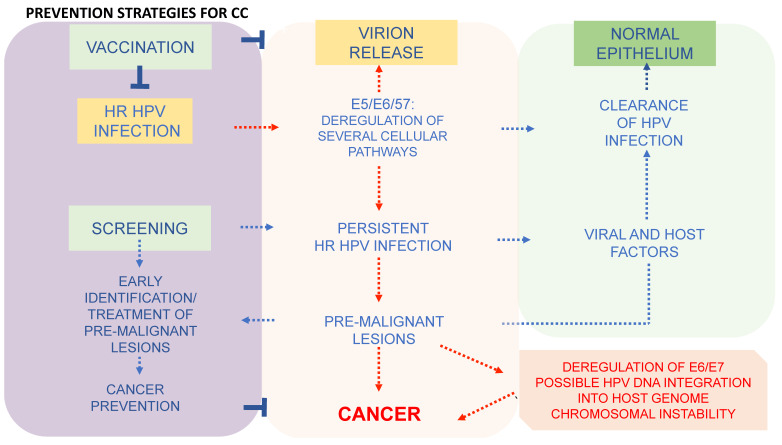
Prevention strategies to control cervical cancer involve both vaccination and screening.

In addition to vaccination, to reduce the incidence of CC, a secondary prevention strategy is necessary and consists of screening programmes based on a HPV DNA test, cervical cytology (Papanicolau test or Pap test) and a triage of women who have tested positive for HR HPVs to identify and treat cervical lesions [119] (Figure 4). As shown in Figure 4, both the primary and secondary preventive strategies are equally important to reduce the CC burden. Preventive strategies act synergistically by targeting the different steps of viral infection in women of different ages [120].

Since the introduction of national CC screening programmes, a constant reduction of the incidence of cancer has been observed in developed countries [13,113]. For decades, CC screening has been only based on the Pap test, which is able to identify abnormal cells among the exfoliated cells of the uterine cervix (Pap smear sample), and this has significantly contributed to reducing the rates of CC cases [119,120].

The current guidelines recommend transitioning from the Pap test to the HPV-DNA test as the primary test for CC screening due to the high sensitivity of the HPV DNA test in order to identify women at risk of developing high-grade cervical lesions (≥CIN2+) and cancer [121,122,123,124]. This change has been achieved in several European countries including Italy for women over 30 years of age [124,125]. Numerous molecular methods have been developed and validated to detect HPV DNA in cervical specimens [126]. However, the Pap test is still used in the triage of women who tested positive for HR HPV-DNA.

The WHO presented a global strategy initiative to accelerate the elimination of CC as a public health problem by 2030 [127]. This strategy provides that every country must achieve and maintain a CC incidence rate below the threshold of 4 cases per 100,000 women per year. To achieve this goal, it is required that (i) 90% of girls should be vaccinated against HPV by the age of 15 years, (ii) 70% of women should undergo screening with a high-performance test at least twice in their lifetime (by the age of 35 and again by the age of 45 years) and (iii) 90% of women identified as having premalignant/malignant cervical lesions should be treated [127].

In contrast to CC, there are no well-defined international guidelines for anal and penile cancer screening [128,129,130] and no approved test to detect HPV-DNA in this setting. However, urethral and anal cytology and a high-resolution anoscopy are highly recommended for some high-risk groups (e.g., PLWHIV and men who have sex with men) (https://www.cdc.gov/std/hpv/stdfact-hpv-and-men.htm, accessed on 11 December 2023). In addition, no approved screening guidelines are available yet for HPV-driven head and neck cancers [100,131].

## 7. Conclusions

Since the discovery of human oncogenic viruses, both functional and epidemiological studies have increased our knowledge of their etiological role in human cancers, thereby allowing for the generation of preventive, diagnostic and therapeutic strategies. It is likely that the role of other infectious agents in human carcinogenesis will be discovered in the near future.

Mucosal alpha HR HPV genotypes are clearly associated with CC in women and with anogenital cancer and a subgroup of HNSCC in both sexes. Several vaccines and screening strategies are currently available worldwide for the prevention of HPV-related CC, while more efforts should be made to increase our knowledge of other HPV-related cancers. The lessons learned from HPV studies indicate that all preventive measures against HPV contributed to the global reduction in the incidence and mortality of HPV-related cancers. Therefore, a strong commitment is now needed at the global level to provide healthcare systems with adequate funding to implement screening programmes and to increase vaccination coverage of the target populations, especially in countries where the incidence of CC is still high.

## Figures and Tables

**Table 2:** Group 1 viruses linked to human cancers are listed with their oncoproteins and the main host targets. The target proteins have been color-coded to highlight that different viruses can target the same protein [42].

VIRUS	ONCOPROTEIN	HOST TARGET
**HPV**	**E6**	**p53**, **mTOR**, **hTERT**
	**E7**	**pRB**
	**E5**	**EGFR**, **ET1**, **COX-2**
**HBV**	**HBx**	**p53**, **pRB**, **Wnt**, **Src**, **DNMTs**, **Ras**, **PI3K**, **JNK**, **NF-κB**, ***ERK***, **TGFβ**, ***HDACs***
**HCV**	**Core**, **NS3**, **Ns5A**	**p53**, **PARP**, **hTERT**, **TGFβ**, ***HDACs***
**EBV (HHV-4)**	**EBNA-1**	
	**LMP-1**	**NF-κB**
	**LMP-2**	**PI3K**, **AKT**, **mTOR**, ***ERK***
**KSHV (HHV-8)**	**Vflip**	**NF-κB**, ***CREB***, **PI3K**, **DDR**
	**LANA**	**p53**, **pRB**, **HIF**, **Notch**, **Wnt**
	**Vgpcr**	**PI3K**, **AKT**, **mTOR**, ***ERK***, **p38**, **JNK**, **NF-κB**
	**Virf-1**	**αIFN**, **p53**, **ATM**, **Bim**
**HTLV-1**	**Tax**	**NF-κB**, ***CREB***, **PI3K**, **DDR**
	**HBZ**	**c-jun**, **E2F**
